# The Translation Factor eIF6 Is a Notch-Dependent Regulator of Cell Migration and Invasion

**DOI:** 10.1371/journal.pone.0032047

**Published:** 2012-02-14

**Authors:** Dario Benelli, Samantha Cialfi, Michela Pinzaglia, Claudio Talora, Paola Londei

**Affiliations:** 1 Department of Cellular Biotechnologies and Haematology, University of Rome Sapienza, Rome, Italy; 2 Department of Molecular Medicine, University of Rome Sapienza, Rome, Italy; 3 Department of Pediatrics and Infantile Neuropsychiatry, University of Rome Sapienza, Rome, Italy; University of Louisville, United States of America

## Abstract

A growing body of evidence indicates that protein factors controlling translation play an important role in tumorigenesis. The protein known as eIF6 is a ribosome anti-association factor that has been implicated in translational initiation and in ribosome synthesis. Over-expression of eIF6 is observed in many natural tumours, and causes developmental and differentiation defects in certain animal models. Here we show that the transcription of the gene encoding eIF6 is modulated by the receptor Notch-1, a protein involved in embryonic development and cell differentiation, as well as in many neoplasms. Inhibition of Notch-1 signalling by γ-secretase inhibitors slowed down cell-cycle progression and reduced the amount of eIF6 in lymphoblastoid and ovarian cancer cell lines. Cultured ovarian cancer cell lines engineered to stably over-expressing eIF6 did not show significant changes in proliferation rate, but displayed an enhanced motility and invasive capacity. Inhibition of Notch-1 signalling in the cells over-expressing eIF6 was effective in slowing down the cell cycle, but did not reduce cell migration and invasion. On the whole, the results suggest that eIF6 is one of the downstream effectors of Notch-1 in the pathway that controls cell motility and invasiveness.

## Introduction

The notion that the control of gene expression at the level of translation is of considerable importance in tumorigenesis is relatively new since several proto-oncogenes and tumour suppressors have been shown to directly regulate ribosome production or translation initiation altering the global translation rate and inducing the translational enhancement or repression of specific mRNAs [1]. The main mechanisms determining the pathological perturbation of translation act at the level of a small set of protein factors regulating translational initiation. Some of them, like eIF2 and eIF4E, are relatively well-characterized [2], [3], [4]. Moreover, certain pathways that control ribosome biogenesis have also been associated with the transformation process. For example, loss of, or functional alterations in, the two major tumor suppressor proteins pRB and p53 cause an up-regulation of ribosome biogenesis in cancer tissues [5]. Likewise, depression of general translation in transgenic mice haploinsufficient for ribosomal protein L24 suppresses the tumor-promoter activity of c-myc [6]. Recently, another translational factor termed eIF6 has been identified as an important player in translational regulation and cell-fate determination. eIF6 is a highly conserved protein shared by Eukaryotes and Archaea that interacts with the large ribosomal subunits regulating the formation of active 80S monosomes [7], [8]. After its initial identification as a ribosome anti-association factor, genetic experiments in yeast led to its reclassification as a factor critically involved in nucleolar rRNA processing and hence in the biogenesis of 60S subunits [9]. Recent experiments in mammals, including the production of eIF6 knock-out transgenic mice, have however demonstrated that eIF6 has indeed a crucial role in translation regulation, possibly in addition to a function in ribosome synthesis [10]. Homozygous ablation of eIF6 determines early lethality in mice embryos; heterozygous mice are, however, viable, although having a reduced rate of protein synthesis. Remarkably, eIF6 haplo-insufficient mice are resistant to myc-induced lymphomagenesis [11]. In line with this result, eIF6 mRNA and protein overexpression has been observed in various natural tumors [12], [13]. In addition to a possible role in tumorigenesis, eIF6 may be important in development and cell-fate determination, as demonstrated by the fact that its altered expression affects the development of *X.laevis*, presumably inhibiting apoptosis [14], [15].

In agreement with such apparent functional complexity, eIF6 expression appears to be highly variable between tissues and even between individual cells in a tissue, with the highest levels observed in epithelia and the lowest in muscle [16]. However, the factors controlling eIF6 expression have not been studied in depth. Intriguingly, the eIF6 promoter lacks a TATA box and contains several GpC islands, features typical of housekeeping genes. Earlier studies also provided evidence for the presence of serum-responsive and NF-kB responsive elements, which were not characterized further [16]. The hitherto best-characterized regulator of eIF6 expression is the GABP complex, a global regulator of ribosome synthesis [17]. In this work we set out to identify additional pathways involved in the transcriptional control of the eIF6 gene. We found that eIF6 transcription is under the control of the transmembrane receptor Notch-1, a protein involved in a wide variety of human neoplasms [18], as well as in embryonic development and cell differentiation [19]. In particular, we demonstrate that eIF6 is a direct transcriptional target of Notch-1 and that the control is performed, at least in part, through RBP-Jk, a downstream modulator of Notch signaling. Inhibition of Notch-1 signaling by γ-secretase inhibitors slowed down cell-cycle progression and decreased the level of eIF6 mRNA in cultured cells. Remarkably, over-expression of eIF6 in stably transformed cell lines had little or no effect on cell proliferation, but increased markedly cell migration and invasion. On the whole, the results suggest that eIF6 is an important downstream effector whereby Notch-1 modulates cell motility and invasivity in physiological or pathological conditions.

## Results

### Notch1 inhibition promotes down-regulation of eIF6 expression in leukemic T cell lines

Overall, the eIF6 gene spans 5,520 base pairs and includes 6 introns and 7 exons. The starting ATG is located in the second exon, while the first exon only contains 5’UTR elements. According to the Ensemble database (ENST00000374450), the main transcription start site of the eIF6 gene is located 498 nucleotides upstream of the initiator ATG. To identify putative transcriptional control elements, we screened about 2000 nucleotides (nt) upstream of the eIF6 gene transcription start site using the Genomatix software. Among others, we found two sequences (G/TGGGAA) at position −1464 and −659 that fully matched the consensus-binding site of the factor CSL (also termed RBP-Jk and CBF-1 in mammals and Suppressor of Hairless [Su(H)] in Drosophila), a known downstream effector of Notch-1 signaling. Indeed, the best characterized mechanism by which Notch activation controls transcription is by converting the DNA-binding protein RBP-Jk from a transcriptional repressor into an activator [20]. This finding suggested that the Notch signaling pathway could be involved in the transcriptional control of the eIF6 gene, a particularly interesting possibility in the light of the fact that Notch signaling has a pivotal role in development, differentiation and proliferation.

**Figure 1:**
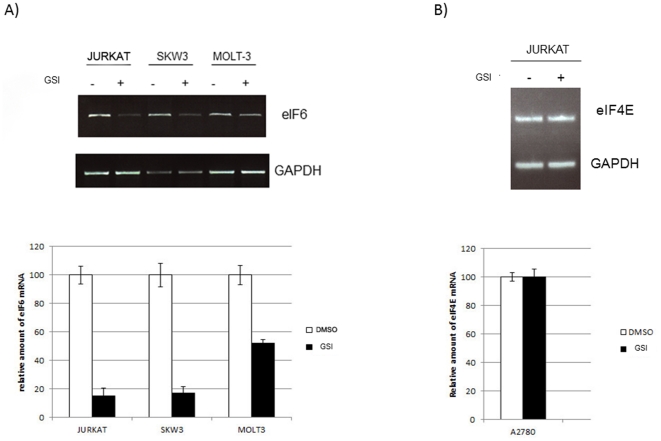
Effect of GSI treatment on the expression of eIF6 and eIF4E. The different T-cell leukemia derived cell lines were treated with 5 µM DAPT or control DMSO for 16h. Total RNA was isolated and expression levels of eIF6 (A) or eIF4E mRNA (B) were estimated by RT-PCR. The intensity of the relevant bands was normalized with respect to the control GAPDH. The histograms represent the average of three independent experiments.

To analyze the role of Notch signaling in the expression of eIF6, three T-Cell leukemia-derived cell lines (Jurkat, SKW3 and MOLT-3) known to carry gain-of-function mutations in Notch-1 [21] were treated with γ-secretase inhibitors (GSI). Γ-secretases trigger Notch signaling by cleaving and releasing the intracellular domain of Notch (ICN), which then translocates to the nucleus where it activates transcription. As shown in Fig. 1, treatment of all three T-ALL cell lines for 16 hours with GSIs decreased significantly the amounts of eIF6 mRNA, indicating that the eIF6 gene is indeed a downstream target of Notch signaling.

To determine whether Notch-1 also influenced the expression of other translation initiation factors known to be involved in translational control and tumorigenesis, the mRNA levels for eIF4E were analyzed in Jurkat cells in the presence and in the absence of GSI. As shown in Fig. 1B, Notch-1 inhibition did not affect the transcription of eIF4E, suggesting that the Notch receptor is not generally involved in regulating the expression of translational factors.

### Notch1-dependent expression of eIF6 involves direct DNA-Binding of Notch1/RBP-jk to CSL elements in the eIF6 regulatory region

**Figure 2:**
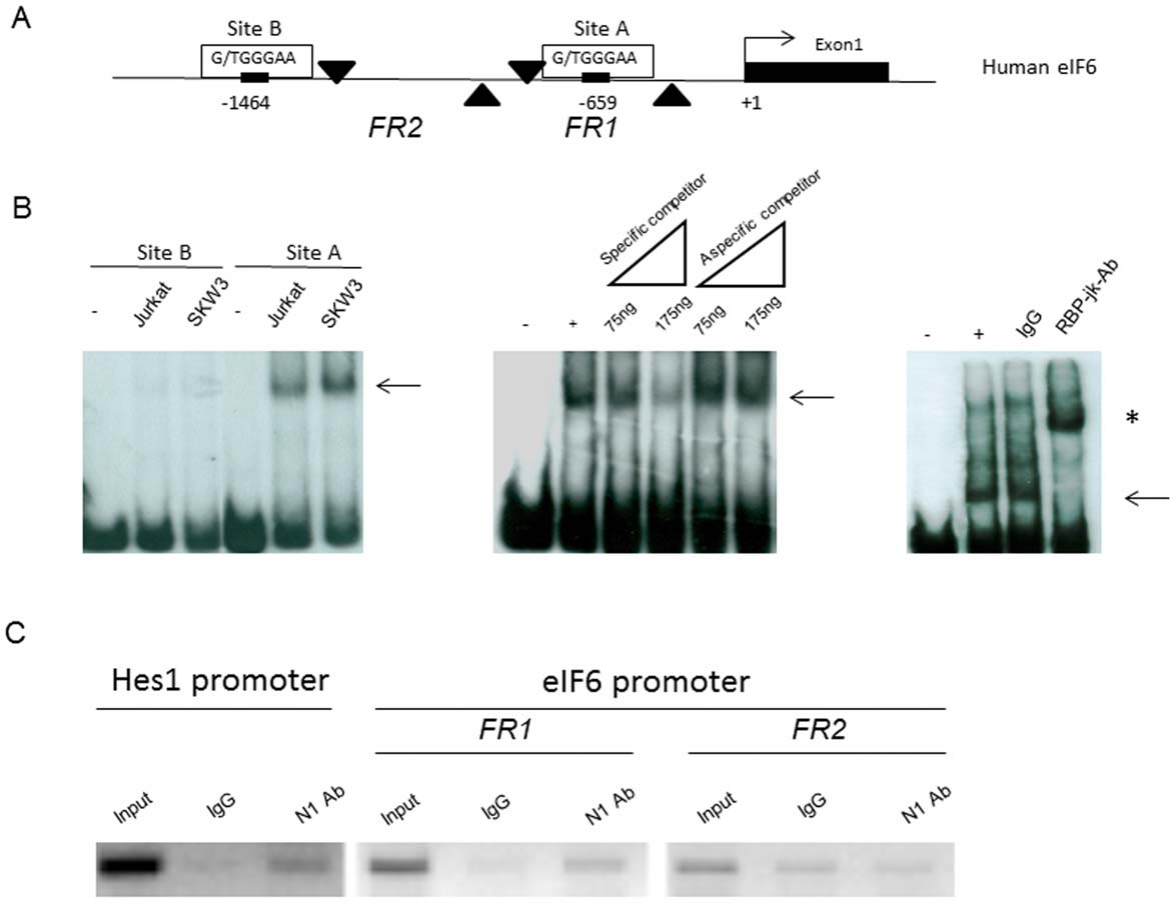
Analysis of RBP-Jk-binding sites in the human eIF6 promoter. (A) Shown is a diagram of the human eIF6 gene 5′-flanking region upstream from the first exon. The positions of putative RBP-Jk-binding sites (sites A–B) and of the PCR primers used in ChIP experiments (FR1 and FR2) are indicated (black arrows). (B) EMSA were performed using nuclear extracts of the indicated cells and the labeled promoter fragment probes. The arrow in all three panels indicates the position of the shifted site A fragment; the asterisk in the third panel indicates the position of the supershift resulting from the addition of anti-RBP-jk antibodies. (C) ChIP assays. Chromatin from Jurkat cell extracts was immunoprecipitated with anti-Notch1 antibodies (N1 Ab) or with control rabbit IgG. The IPs were analyzed by PCR using primers specific for the indicated promoter regions of eIF6 or for the Notch-binding region of the Hes1 promoter as the positive control.

As stated above, the eIF6 promoter contains two putative binding sites for RBP-Jk, a well-known downstream effector of the “canonical” Notch signaling pathway (Fig. 2A). To address whether Notch1/RBP-Jk directly associated with the eIF6 promoter, an electrophoretic mobility shift assay (EMSA) was performed incubating nuclear extracts of Jurkat cells with fragments of the eIF6 promoter.

The results, shown in Fig. 2B, revealed a clear shift only for the fragment containing the −659 putative RBP-Jk-binding site. The shift was abolished by the addition of a 100/200-fold excess of unlabeled probe, but not by the addition of a 100/200 fold excess of non-specific unlabeled probe. Pre-incubation of the cell lysates from Jurkat cells with RBP-Jk specific antibodies produced a super-shift that revealed the presence of RBP-Jk in the complex (Fig. 2B).

To further validate the above findings, chromatin immunoprecipitation (ChIP) assays were performed using anti-Notch-1 antibodies on Jurkat cell extracts. As Fig. 2C illustrates, the specific antibodies, but not non-specific IgGs, were able to immunoprecipitate chromatin enriched in eIF6 promoter sequence containing the RBP-Jk-binding element at position −659. In agreement with the EMSA assays, the region of eIF6 promoter between the RBP-Jk-binding elements was not enriched in the immuno-precipitates.

### Activated Notch1 stimulates eIF6 promoter activity through an RBP-Jk-dependent mechanism

**Figure 3:**
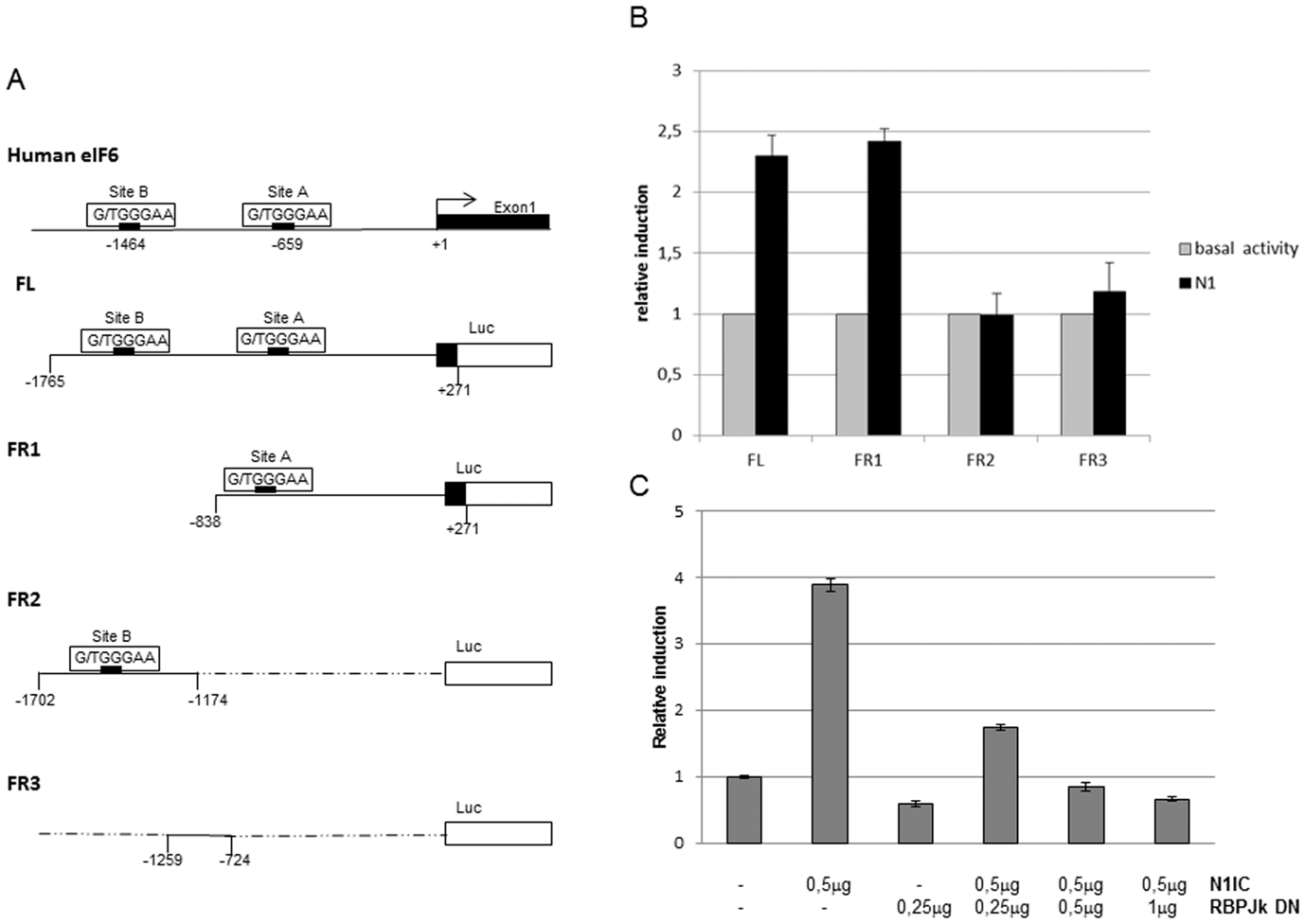
Luciferase reporter assay. (A) Schematic representation of the region of the human eIF6 promoter containing two putative RBP-jk-binding sites (sites A-B). Different tracts of the promoter were cloned upstream of the luciferase gene in the pGL3 Basic luciferase plasmid obtaining the constructs indicated as FL, FR1, FR2 and FR3. (B) Luciferase assay. NIH-3T3 cells were cotransfected with *Renilla* luciferase plasmid and one of the reporter plasmids shown in A in the presence (black columns) or the absence (grey columns) of a plasmid expressing human Notch-1 (N1). (C) NIH-3T3 cells co-transfected with the FL plasmid and the N1 plasmid were further transfected with increasing amounts (0,25-0,5-1 µg) of a plasmid expressing a dominant-negative form of RBP-jk (RBP-jk DN).

The mechanism of Notch-1 transcriptional regulation of the eIF6 gene was further investigated by performing luciferase reporter assays. To this end, reporter plasmids were constructed in which different fragments of the human eIF6 promoter (−1765 to +227) were cloned upstream of the firefly luciferase gene (Fig. 3A). Each of the reporter constructs was transfected in NIH3T3 cells either without or with simultaneous co-transfection with a plasmid expressing activated Notch-1 (N1). As shown in Fig. 3B, co-transfection of eIF6-Luc/full-length (containing the −1765 to +227 promoter region including both the identified putative RBP-Jk-binding elements) and activated Notch-1 resulted in a about 2.5-fold increase in luciferase activity over the level attained in absence of Notch-1. Similar levels of Notch-1 transcriptional stimulation were achieved with a reporter construct containing only the RBP-Jk-binding element positioned at −659 bp, further indicating that the distal putative RBP-Jk-binding site is not involved in transcriptional modulation. Accordingly, the construct containing only the −1464 RBP-Jk-binding element was completely unresponsive to Notch-1. Similar negative results were obtained with the reporter construct containing the region between the RBP-Jk-binding elements. As anticipated by the EMSA experiments, the transcriptional stimulation of the eIF6 promoter by activated Notch-1 was suppressed by the expression of a dominant-negative form of RBP-Jk [22] which is unable to bind DNA but is able to interact with Notch1 (Fig. 3C).

### eIF6 over-expression enhances cell migration and invasiveness without affecting proliferation

**Figure 4:**
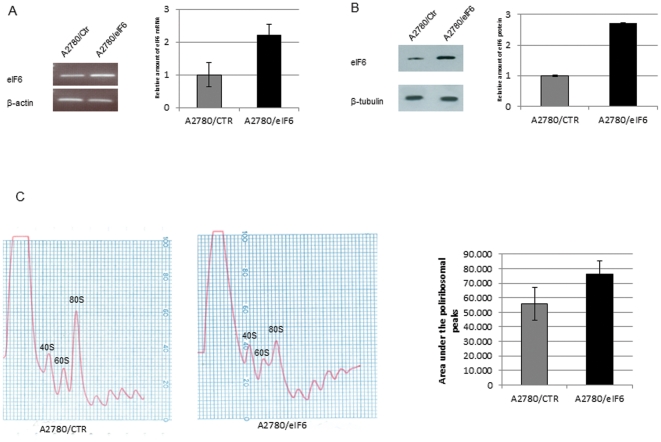
eIF6 expression in stably-transfected A2780 ovarian cancer cells. eIF6 expression in a pool of A2780 cells stably transfected with the pcDNA3-eIF6 plasmid was analyzed by RT-PCR (A) and western blotting (B) as indicated. The intensity of the eIF6 RNA and protein bands was quantified relative to β-actin and β-tubulin, respectively, using the ImageJ software. The results represent the average of three independent experiments. (C) Analysis of the polysomal profiles of A2780/eIF6 and control cells by density gradient centrifugation. The areas under the polysomal peaks were quantified using the ImageJ software.

The data reported above suggested that eIF6 acted as a downstream mediator of Notch-1 signaling. It is well established that Notch signaling, besides being important for development and differentiation, promotes survival and proliferation of many types of cancer cells. For instance, T-ALL cells as well as ovarian cancer cells are characterized by activated Notch signaling [21], [23], [24]; accordingly, down-regulation of Notch-1 by GSI contributes to cell growth inhibition and apoptosis in ovarian cancer cells [23]. Notably, over-expression of eIF6 has been observed in a significant proportion of ovarian serous adenocarcinomas and hematological cancers [25]. To test whether the Notch-dependent stimulation of cellular proliferation could be attributed, at least in part, to up-regulation of eIF6 expression, A2780 ovarian cancer cell lines were transformed with a plasmid expressing eIF6 from a strong promoter. As shown in Fig. 4A/B, eIF6 stable clones had on average 2-3-fold higher levels of eIF6 with respect to the controls, whereas no clone producing the factor in really massive quantities could be isolated, suggesting that a large excess of eIF6 is toxic for the cells. Analysis of the polysomal profiles of the selected transformants (Fig. 4C) showed a decreased 80S peak, consistent with the described anti-association activity of eIF6 on ribosomal subunits [8]. However, the decrease of the 80S peak was accompanied by an increase in the total amount of polysomes (Fig. 4C), suggesting that a 2–3 fold over-expression of eIF6 had a stimulatory effect on protein synthesis.

**Figure 5:**
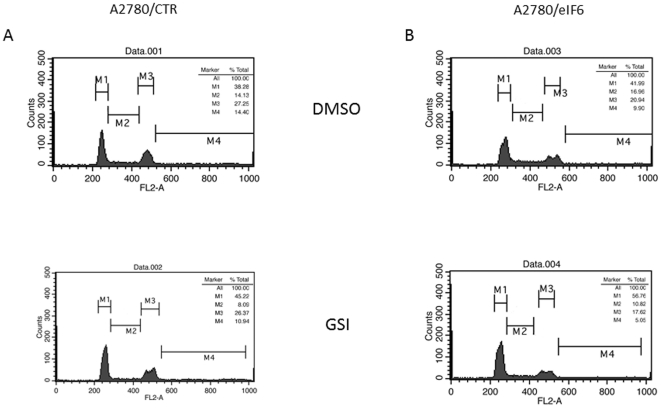
eIF6 over-expression does not significantly affect cell cycle. FACS analysis of cell-cycle distribution of control (A) and eIF6-over-expressing (B) A2780 cells grown for 72 h in the absence (top) or in the presence (bottom) of 75 µM DAPT.

To investigate the impact of excess eIF6 on proliferation, control and eIF6-expressing A2780 cells were cultured for 72 hrs with or without added GSI and were then subjected to cell cycle analysis. Without added GSI, both control and eIF6-expressing cells had a similar distribution along the cell cycle, indicating that the presence of excess eIF6 did not significantly affect proliferation (Fig. 5, upper panels). Treatment with GSI of A2780-pCDNA3 control cells caused a dose-dependent induction of cell-cycle arrest, evidenced by an increased proportion of cells in G1 phase and a decrease in the proportion of cells in S phase. A similar arrest in G1 following GSI treatment was also observed for the A2780-eIF6 cells, indicating that eIF6 is not sufficient to rescue the GSI induced cell cycle arrest (Fig. 5, bottom panels). We also performed colony-forming assays and found that clonal growth of A2780 cells was not altered by eIF6 expression (data not shown).

In addition to affecting proliferation, the Notch pathway has been reported to regulate motility and invasiveness of cancer cells [26] Accordingly, we tested whether eIF6 overexpression had any impact on the migratory and invasive capabilities of the A2780 cells, and whether these properties were affected by Notch inhibition. To this end, trans-well migration and invasion assays were performed with A2780 pCDNA3 control cells and A2780-eIF6 cells, with and without treatment with GSI.

**Figure 6:**
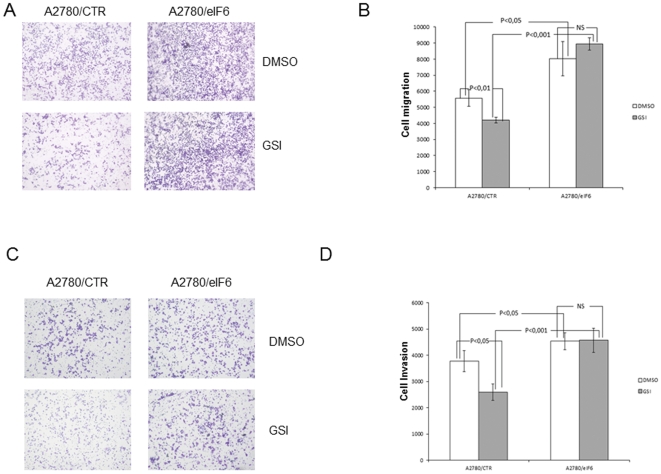
Over-expression of eIF6 enhances migration and invasivity of ovarian cancer cells. (A, B) Migration assay: A2780/eIF6 and control cells were treated with DAPT 75 µM or DMSO for 36 h then seeded in the upper side of migration chambers. The cells migrated to the lower chambers after 36 h of incubation were stained with crystal violet dye. (C, D) Invasivity assay: cells were treated with DMSO or DAPT 75 µM for 36 hours and seeded in the upper side of invasion chambers. After 36 h, cells migrated in the lower chamber were stained. The total stained area in the lower chambers was estimated using the Image-J software. The histograms in (B) and (D) represent the average of three independent experiments. P values estimating the statistical significance of the observed experimental variations between different data sets (control cells with and without GSI; eIF6 cells with and without GSI; control and eIF6 cells without GSI; control and eIF6 cells with GSI) are shown for both cell migration and invasion experiments.

As shown in Fig. 6 (A,B), the A2780-eIF6 cells displayed a markedly higher (about 40%) migratory capacity with respect to the A2780-pcDNA3 cells, suggesting that over-dosage of eIF6 enhanced cell motility. Similar results were obtained when the cells' invasive capacity was tested by trans-well/matrigel assays (Fig. 6 C,D): the A2780-eIF6 cells were about 20% more invasive than the controls. That eIF6 indeed promoted a migratory and invasive phenotype was confirmed by GSI-inhibition assays. Strikingly, while the control cells showed, as expected, a reduction in their capacity both to migrate to the bottom well and to degrade the matrigel layer (25–35% compared to the DMSO), the A2780-eIF6 cells were completely unaffected by GSI and remained highly mobile and invasive (Fig. 6). Overall, the results suggest that eIF6 is implicated in the control of cell motility/invasiveness, and is one of the downstream effectors whereby Notch signaling modulates these cellular properties.

## Discussion

Translation, long regarded as a rather dull cellular housekeeping activity, is being increasingly recognized as an important checkpoint for modulating many cellular functions. Indeed, it has been known for some time that certain translational factors, notably eIF4E and eIF2, are downstream targets of various signaling pathways that control cell proliferation [3], [27], [28]. Under certain circumstances, an altered functioning of these factors can steer the cell towards tumoral transformation. Recently, the protein termed eIF6 has been added to the list of the translation factors possibly involved in the control of cell proliferation. eIF6 is a 27 kDa monomeric protein, first identified as a ribosome anti-association factor [7] and later also implicated in the control of ribosome synthesis [9]. Although eIF6 is an essential factor and its deletion causes early embryonic lethality [10], its precise function in translation (or in other cellular functions) remains elusive.

We show here that the transcription of the eIF6 gene is regulated by the Notch-1 receptor, controlling an evolutionarily conserved signal transduction pathway of paramount importance in development, cell differentiation and proliferation [20]. Alterations in the Notch-1 pathway underlie many developmental and differentiation defects, and are also observed in several types of tumors [18]. We found that stable expression of eIF6 does not significantly alter the rate of cellular proliferation, but has a profound effect on cell motility/invasiveness. In A2780 ovarian cancer cell lines, a 2–3 fold over-expression of eIF6 enhances the cell invasive properties, and is moreover sufficient to rescue completely the inhibition of migration and invasiveness caused by treatment with GSI, suggesting that eIF6 is a main downstream target of Notch signaling in the pathway controlling cell motility/invasiveness. These results agree with previous data showing that over-expression of eIF6 affects morphogenesis during *D.melanogaster* development [29], that eIF6 phosphorylation and its association with the cytoskeleton are developmentally regulated in *X.laevis* [30], and that eIF6 is frequently over-expressed in several types of metastatic tumors [13]. Therefore, our data indicate the possibility that Notch/eIF6 axis could be responsible for the triggering, at least in part, of metastatic mechanisms in the invasive cells.

It is noteworthy that only modest variations of the eIF6 cellular levels seem to be able to significantly affect cell behavior. As we show in this work, Notch-1 activation increases the transcription of the eIF6 gene 2–3 fold. Induced over-expression of the protein within a similar order of magnitude is sufficient to markedly enhance cell motility and capacity to migrate through a matrigel layer.

The mechanism whereby eIF6 affects cell motility/invasiveness remains to be elucidated. The established function of eIF6 is on protein synthesis and/or ribosome synthesis, but it is likely to act on other cellular functions as well. Indeed, eIF6 has been shown to bind to beta4-integrin and to be present at hemi-desmosomes, suggesting a participation in cell-cell adhesion and communication [31], [32]. These cytoskeletal connections of eIF6, whose functional significance is still unknown, are prime candidates to explain the influence of the factor on cell migration and invasion. The alternative possibility is that eIF6 may control, directly or indirectly, the synthesis of some protein(s) involved in the control of cell motility. For instance, abundance or dearth of eIF6 might favor (or otherwise) the translation of specific mRNAs encoding products important for cell motility. Some support for this hypothesis comes from our observation that the A2780 cells over-expressing eIF6 have a lesser amount of 80S ribosomes but an increased amount of polysomes (Fig. 4). This suggests that a 2–3 fold increase in eIF6, as observed in this study, might exert a stimulatory effect on protein synthesis, either by promoting initiation or by accelerating ribosome recycling. Such an effect could in turn increase (or decrease) the translation rate of specific mRNAs.

A still more intriguing possibility is that eIF6 controls the abundance and/or translation of certain mRNAs by a microRNA-mediated mechanism. In fact, it has been reported that eIF6 associates with the RISC and, in certain organisms at least, is required for miRNA-mediated control of translation [33]. Much more research is required to fully elucidate the cellular functions of this very interesting factor.

## Materials and Methods

### Plasmids

Human eIF6 promoter (−1754 to +227) was PCR amplified using a proof reading Taq (Kapa HiFi) with the following primers sense 5′-GGACCTCATCACCAAGTATC-3′ and antisense 5′-CTGTACCTTGGACTCCCTAA-3′. The amplified product of 1992 bp was cloned by TA-vector kit (RBC Bioscience) and successively inserted into pGL3 Basic luciferase plasmid (Promega) using the *Xho*I*-Hind*III restriction sites. This luciferase reporter construct, termed *FL eIF6 prom-luc*, was then used to generate the subsequent three constructs for luciferase assays using the primers summarized in Table 1.

**Table 1:** List of the primers used for the cloning of different constructs.

Name construct	Primers
***FR1***	
Forward	5′-CATGGTATGCTCGAGGCTGTGACAGGTTGTGGGCC-3′
Reverse	5′-CCCAAGCTTCTGTACCTTGGACTCCC-3′
***FR2***	
Forward	5′-CATGGTATGCTCGAGCCTGGGGGTCCCAAGGG-3′
Reverse	5′-CCCAAGCTTCTCCAGGACTCTCTGCCC-3′
***FR3***	
Forward	5′-CATGGTATGCTCGAGCTGCTGGAGAAGGGGTCAG-3′
Reverse	5′-CCCAAGCTTAGGATACACCAGGCGCTG-3′

The reaction sites used for the cloning are underlined.

To clone the human full-length eIF6 gene we used the following primers: forward (5′-CCCAAGCTTCTGGTTACTTGGCCTCAT-3′) and reverse (5′-CATGGTATGCTCGAGAATGTGGAGAAGGTTGGC-3′). The resulting amplification product was inserted at the *Hind*III*-Xho*I sites of pcDNA™ 3.1(+) vector. All plasmids were verified by DNA sequencing.

### Cell transfection and luciferase assay

Transient transfection experiments were performed by Lipofectamine 2000 kit (Invitrogen) according to manufacturer's instructions. NIH/3T3 cells, grown in DMEM supplemented with 10% FBS and L-Glu 2%, were seeded in 24-well plates at a density of 3×10^5^ cells per well. The day after cells were transfected with the *eIF6* promoter vectors (−1754 to +227) as reporter (0.5 µg) in presence or absence of the expression vector for human Notch1 (1 µg), previously described (Talora, C. *et al*., 2002) as effector gene and pRL-CMV vector (10 ng) expressing *Renilla* luciferase. pcDNA_3_ vector was used as an empty control vector and was added to each sample ensure an equal amount of total DNA.

The day after the cells were lysed using Dual-Luciferase/Renilla Reporter Assay System (Promega, Madison, WI) reagents in accordance with the manufacturer's instructions.

Firefly- and pRL-TK-derived Renilla luciferase activities were measured in each sample using a Triathler Multilabel Tester (Beijing Huaruison Science and Technology Development Co., Ltd).

### Polysomal profiles

A2780 and the corresponding stable eIF6 clone cells (about 5×10^6^ cells) were treated with cycloheximide (CHX) to a final concentration of 100 µg/ml and then incubated at 37°C for 15 min. After washing the monolayer once with ice-cold PBS 1X+CHX (50 µg/ml), the cells were scraped in 500 µl of ice-cold lysis buffer (10 mM Tris-HCl pH 7.4, 10 mM KCl, 15 mM MgCl_2_, 1 mM DTT, 1% Triton-X 100, 1% deoxycholate, 0,5 unitsµl^−1^ rRNasin, 100 µg/ml CHX) for 5 min on ice. Cell debrises were removed by a 8 min centrifugation at 10,000 g at 4°C. 30 A_260_ units of supernatants were layered on top of a linear 15–50% (w/v) sucrose gradient containing 20 mM Tris-HCl pH 7.4, 5 mM MgCl_2_, 140 mM KCl, 0,5 mM DTT and 0,1 mg/ml CHX. The gradients were centrifuged at 4°C in a SW41 Beckman rotor for 2 h at 39,000 rpm and unloaded while monitoring absorbance at 260 nm with the ISCO UA-5 absorbance instrument. Successively, the graphic of polyribosomal profiles was analyzed with the ImageJ software in order to calculate the area under the peaks of interest.

### Chromatin Immunoprecipitation (ChIP)

Protein complexes were cross-linked to DNA in living nuclei by adding formaldehyde (Sigma, Inc.) directly to fresh Jurkat cell lines to a final concentration of 1%. Crosslinking was allowed to proceed for 10 min at 37°C and was then stopped by the addition of glycine to a final concentration of 0.125 M. Cross-linked cells were washed twice with phosphate-buffered saline and pelletted. Nuclei were extract with a buffer containing 10 mM Tris pH 8, 0,25% Triton-X 100, 10 mM Na-EDTA, 0.5 mM Na-EGTA and protease inhibitors, pelleted by microcentrifugation and lysed by incubation in SDS lysis buffer (0.5% sodium dodecyl sulfate, 5 mM Na-EDTA, 50 mM Trischloride pH 8), containing protease inhibitors. The resulting chromatin solution was sonicated for 15 pulses of 15 s at 80% power to generate 300–1000 bp DNA fragments. After microcentrifugation, the supernatant was diluted 1∶5 with a dilution buffer (0.01% sodium dodecyl sulfate, 1% Triton X-100, 1 mM EDTA, 20 mM Tris-chloride pH 8, 150 mM NaCl, containing protease inhibitors), and aliquoted. After precleaning with Salmon Sperm DNA/Protein A agarose (Upstate Biotechnology) 5 µg of antibodies anti-Notch-1 (C-20) or normal rabbit IgG, (Santa Cruz Biotechnology Inc.) were added to each aliquot of chromatin and incubated on a rotating platform for 12–16 h at 4°C. Antibody–protein–DNA complexes were isolated by immunoprecipitation with Salmon Sperm DNA/Protein A agarose (Upstate Biotechnology). Following extensive washing, bound DNA fragments were eluted and analyzed by subsequent PCR with the following primers: CSL fragment forward (5′-CATGGTATGCTCGAGGCTGTGACAGGTTGTGGGCC-3′), CSL fragment reverse (5′-CCCAAGCTTCCACGATGTGCCTCTCGC-3′), intermediate fragment forward (5′-CATGGTATGCTCGAGCTGCTGGAGAAGGGGTCAG-3′), intermediate fragment reverse (5′-CCCAAGCTTAGGATACACCAGGCGCTG-3′).

As the positive control, we performed PCR to amplify a fragment of the Hes1 promoter known to bind Notch-1, using the following primers: Hes1 promoter forward (5′-CTGTGGGAAAGAAAGTTTGGG-3′); Hes1 promoter reverse (5′-GACCAAGGAGAGAGGTAGAC-3′).

### EMSA

The frozen cell pellet was thawed, resuspended at a concentration of 10 µl/10^7^ cells in cold buffer C (Hepes 20 mM pH 7.9, NaCl 0.4 M, EDTA 1 mM, EGTA 1 mM, DTT 1 mM, PMSF + protease inhibitors 1 mM and 1% NP-40) and vortexed vigorously for 2 min at 4°C. Debris was pelleted and the supernatant removed as the whole-cell extract. EMSA were performed by using double strand DNA obtained digesting WT eIF6 prom-luc with AvaI or ApaLI-SpeI. The reaction products, DNA fragments of 164 and 226 bp respectively, contained the CSL elements of our interest. The fragments were purified from agarose gels by DNA Fragment Extraction kit (RBC Bioscience). Successively, these products were labeled with [α^32^P]dCTP by Klenow enzyme (Roche) and purified over a G25 sepharose column. The probe (10 fmol) was incubated for 20 min at room temperature

### Cell lines and treatments

Human T-lymphoblastic cell lines JURKAT, SKW3 and MOLT3 and human ovarian cancer cell line A2780 were kept in culture in RPMI 1640 (Gibco) supplemented with 10% FBS (Gibco) and 1 mmol/L L-glutamine at 37°C in 5% CO_2_. Mouse embryonic fibroblast cells NIH3T3 were kept in culture in D-MEM (Gibco) supplemented with 10% FBS (Gibco) and 1 mmol/L L-glutamine at 37°C in 5% CO_2_. Whenever required, ã-secretase inhibitor IX (GSI) (Calbiochem) or DMSO was added at the concentration of 5ìM to the growth medium of human T-lymphoblastic cell lines JURKAT, SKW3 and MOLT3 for 16 h. In the experiments performed with A2780 cells, GSI was used at the final concentration of 75 µM for 16 h. At the end of incubation the cells were washed with PBS 1X and collected. Total protein extract was obtained by lysing the cells with extraction buffer (20 mM Tris-HCl pH7.5, 150 mM NaCl, 1 mM EDTA pH8, 1% Triton-X and protease inhibitor cocktail) (Roche).

### Stable transfection of the A2780 cell line

16 hours prior to transfection, A2780 cells were seeded at 50% confluence in 60 mm dish. Transfection was carried out according to the manufacturer's instructions of Lipofectamine 2000 (Invitrogen) using 5 µg of linearized human full-length eIF6 expression vector or equal amounts of pcDNA3 as the control. After 24 h, the cells were trypsinized and distributed over three 100 mm dishes per well. Medium was replaced every three days with culture medium containing 200 µg/ml of G418 (selection medium) until clones formed which were large enough to isolate (about two weeks). Selected positive clones were pooled and used for the next experiments.

### Migration assay

Cells were pretreated in complete medium containing 75 µM of GSI for 36 h before plating (2.5×10^5^ per well) in the BD Falcon™ Cell Culture Inserts (BD Biosciences). Mock treatments were carried out pretreating the cells in the same medium with DMSO. The chambers with the cells were placed on 24-well plates containing medium without serum. In the lower chamber, medium supplemented with 10% FBS was used as chemo-attractant. After 36 hours, cells migrated to the lower chamber were stained with crystal violet dye. The total stained area was quantified using the Image-J software. Experiments were carried out in triplicate and repeated three times. Statistical analyses were performed using paired Student's t test for independent samples. Differences were considered significant if the probability (p) was <0.05.

### Invasion in Matrigel-coated chambers

Cells were pretreated in complete medium containing 75 µM of GSI for 36 h before plating (2.5×10^5^ per well) in the BD Matrigel invasion chambers (BD Biosciences). Mock treatments were carried out pretreating the cells in the same medium with DMSO. Cells were seeded in the upper chamber in treatment medium without serum. In the lower chamber, medium supplemented with 10% FBS was used as chemo-attractant After 36 hours, cells migrated to the lower chamber were stained with crystal violet dye. The total stained area was quantified using the Image-J software Experiments were carried out in triplicate and repeated three times. Statistical analyses were performed using paired Student's t test for independent samples. Differences were considered significant if the probability (p) was <0.05

### RT-PCR

Human T-lymphoblastic cell lines JURKAT, SKW3 and MOLT3 were treated in complete medium containing 5 µM of GSI for 16 h. Mock treatments were carried out treating the cells in the same medium with DMSO. Total RNA was extracted from cells using TriZol reagent (Invitrogen). One microgram of RNA was used to generate cDNA using oligo dT (Applied Biosystem) and M-MLV reverse transcriptase (Promega). Two microliters of cDNA was used for PCR reactions. All reactions were carried out for 25 cycles. Primer used for the analysis of eIF6 mRNA levels were as follows: *eIF6-PCR*, forward 5′- CAATGTCACCACCTGCAATG-3′ and reverse 5′-AGTCATTCACCACCATCCCA-3′. The primers used for the analysis of eIF4E mRNA levels were as follows: eIF4E FW 5′- GGAAACCACCCCTACTCCTA-3′ and eIF4E Rev 5′- ATGGTTGTACAGAGCCCAAA-3′.

All PCR products were analyzed on 1% agarose gel electrophoresis. For calculating the expression level, the band intensities of PCR products were measured using ImageJ (NIH) software and normalized to that of GAPDH.

### Flow cytometry and cell cycle analysis

The cell cycle was analyzed by flow cytometry. Cells (1×10^6^) were collected and washed in PBS, then fixed in 75% alcohol for 30 min at 4°C. After washing in cold PBS three times, cells were resuspended in 1 ml of PBS solution with 40 µg of propidium iodide and 100 µg of RNase A for 30 min at 37°C. Samples were then analyzed for their DNA content by FACSCalibur™ (BD Biosciencies). Each experiment was repeated three times.
